# Immunoglobulin A nephropathy associated with acute hepatitis E infection: First case report 

**DOI:** 10.5414/CNCS111100

**Published:** 2023-06-08

**Authors:** Sidra Shafiq Cheema, Manal Fatima Cheema, Samreen Gilani, Shafiqur Rehman Cheema

**Affiliations:** Head Department of Nephrology, Jinnah Hospital Lahore, Usmani Rd, Quaid-i-Azam Campus, Lahore, Punjab, Pakistan

**Keywords:** IgA nephropathy, hepatitis E, nephrotic syndrome, hematuria

## Abstract

In this case report, we describe a young male patient who presented with gross hematuria and nephrotic syndrome a few weeks after serologically positive acute hepatitis E virus (HEV) infection. Histopathological examination of renal core biopsy revealed that the majority of the viable glomeruli had a predominantly mesangiopathic process characterized by mild to moderate diffuse increase in mesangial matrix and cellularity with segmental variation. Immunofluorescence microscopy depicted a strong (3+) granular mesangial and capillary loop staining for IgA, consistent with IgA nephropathy (IgAN). This pattern of mesangial and glomerular capillary loop staining of IgA is suggestive of secondary IgAN. Further research is required to explore the relationship between IgAN and acute HEV infection.

## Introduction 

IgA nephropathy (IgAN) is the most common lesion found to cause primary (idiopathic) glomerulonephritis throughout most high-income countries of the world [1]. There are case reports of IgAN associated with other conditions like HBV, HCV, HAV, HIV, celiac disease, alcoholic liver disease, and a few rheumatological disorders. Over the past two decades, there have been several case reports [1, 2, 3, 4] of its association with acute hepatitis A infection. Glomerular manifestations including “IgAN relapses” are reported among solid organ transplant patients with HEV genotype 3 [5], but no case of IgAN has been reported in immunocompetent patients with HEV. We are presenting a case of a young male patient who developed nephrotic syndrome, hematuria, and acute kidney injury immediately after recovery from acute hepatitis E. 

## Case report 

A 25-year-old male presented to the emergency department with gross hematuria and nephrotic syndrome 3 weeks after severe acute HEV infection. While his past medical history was insignificant, a review of his medical records revealed that ~ 3 weeks prior he had presented to his primary care physician with nausea, fatigue, anorexia, malaise, yellowish discoloration, and abdominal discomfort. On physical examination, he had BP of 100/60 mmHg, pulse rate of 95 beats/minute, respiratory rate of 16/min, and temperature of 37.8 °C/100 °F. Hand, ears, eyes, nose and throat (HEENT) examination was significant for scleral icterus, dry mucus membranes, flat jugular venous pressure (JVP), and poor skin turgor. The abdomen was soft, non-distended with good bowl sounds, but there was slight tenderness in the right upper quadrant. The lower border of the liver was palpable with no splenomegaly. The chest was clear on auscultation (no added breath sounds), and on the precordium exam, first and second heart sounds were audible, and no gallop, rub, or murmur was heard. The rest of the physical exam was unremarkable. 

Laboratory investigations showed: elevated total bilirubin (8 mg/dL), AST level (1,020 U/L), and ALT level (1,750 U/L), whereas alkaline phosphatase, GGT, and serum albumin were within normal range. LDL (146 mg/dL), triglyceride (312 mg/dL), and uric acid (7.8 mg/dL) were raised, but total cholesterol was normal (190 mg/dL). Serum creatinine was 0.9 mg/dL. Additionally, anti-HEV IgM and IgG both were positive. Urine complete examination was unremarkable except for increased specific gravity of 1030, 1+ protein. He was diagnosed with acute HEV infection. Supportive treatment including intravenous hydration was started, and he was discharged from the hospital after 3 days. 

**Figure 1.**
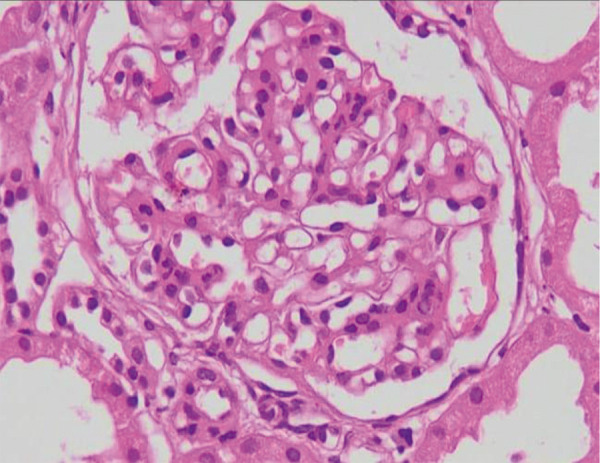
Glomeruli exhibit mild-moderate degree of mesangial hypercellularity and matrix increase; light microscopy (H & E stain, original magnification × 400).

**Figure 2.**
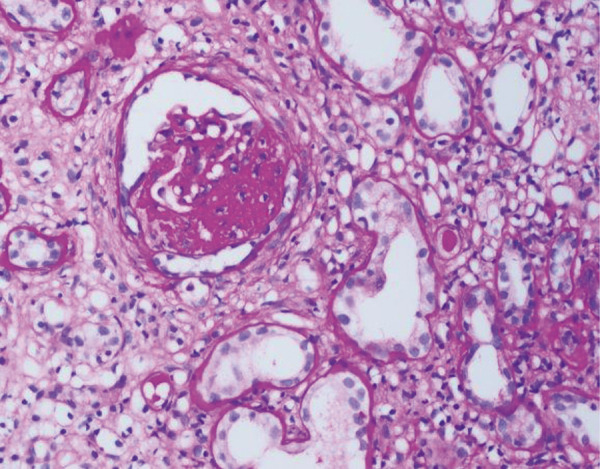
One of the globally sclerosed glomeruli; light microscopy (PAS stain, original magnification × 100).

Approximately 2 and half weeks after discharge, he noticed lower extremities edema and dark-colored urine, and he was referred to nephrology. Physical examination was significant for BP of 145/90 mmHg, pulse rate of 70 beats/min, RR of 12/min, afebrile, two plus edema on bilateral lower extremities. All examinations including chest, cardiac, abdominal, neurological, and skin were unremarkable. The laboratory tests revealed serum creatinine (1.7 mg/dL), albumin (3.2 g/dL), cholesterol (320 mg/dL), urine complete (15 – 20 RBC), and a urine protein-creatinine ratio UPCR (4.7 g/day). Further laboratory investigations showed that antinuclear antibody, antistreptolysin O, complement C3, C4, hepatitis B, hepatitis C, human immunodeficiency virus, and hepatitis A serology were negative. A real-time ultrasound-guided core kidney biopsy was performed. Histopathological examination of the renal core biopsy revealed 10 glomeruli. The majority of the viable glomeruli revealed a predominantly mesangiopathic process characterized by mild to moderate diffuse increase in mesangial matrix and cellularity with segmental variation (Figure 1). Focal tuft adhesion is also noted; however, there was no activity, extra- or endocapillary proliferation, or fibrinoid necrosis. There was focal interstitial edema, infiltrate, and 2 globally sclerosed glomeruli (Figure 2). 

**Figure 3.**
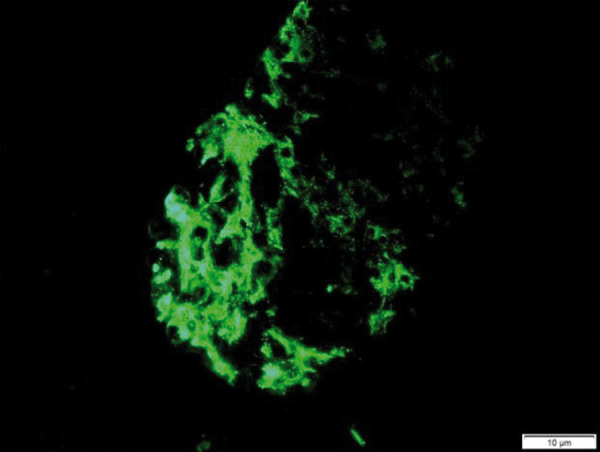
Mesangial and capillary wall IgA deposits (immunofluorescence staining for IgA, original magnification × 400).

Immunofluorescence microscopy depicted a strong (3+) granular mesangial (Figure 3) and capillary loop staining (in a segmental fashion) for IgA. This pattern of mesangial and glomerular capillary loop staining of IgA is suggestive of secondary IgAN. It also depicted 1+ staining for C3 while showing none for IgG, IgM, κ, or λ (Oxford-MEST-C: M1, E1, S1, T0, C0). When IgAN was confirmed on biopsy, the patient was started on prednisone 1 mg/kg body weight at 60 mg/d, valsartan 160 mg/d , furosemide 40 mg once daily, and atorvastatin 20 mg once daily. After 8 weeks of treatment, his creatinine decreased to 1.2 mg/dL, UPCR to 1.2 g/day, and RBCs to 5 – 10 from more than 50/HPF 8 weeks prior. Prednisone was tapered to 40 mg/d. 

## Clinical course and outcome 

He was followed up in the nephrology outpatient clinic, and after 12 weeks of treatment he improved further with BP reduced to 120/78 mmHg, and the lower extremity edema was resolved. His creatinine had decreased to 1.1 mg/dL, UPCR to 0.5 g/day, and RBCs to only 0 – 5/HPF. Total cholesterol dropped to 220 mg/dL, serum albumin increased to 4.1 g/dL. Liver transaminases and bilirubin had already returned to normal range. On follow-up at 6 months on tapering dosages of prednisone, until the time of reporting this case, our patient revealed continued improvement. Subsequently, valsartan has been reduced to 80 mg daily, furosemide has been discontinued, and atorvastatin and prednisone have both been reduced to 10 mg/d. The patient appears to have achieved complete remission with normal kidney functions and UPCR of equal to or less than 500 mg/day. 

## Discussion 

To our knowledge, this is the first case report of IgAN associated with HEV infection. This young patient, with no prior history of hepatitis or kidney disease, presented with sudden-onset nephrotic syndrome, gross hematuria, and acute kidney injury immediately following or simultaneously with an acute HEV infection. In addition to hepatitis B & C, secondary IgAN is also caused by chronic liver disease, especially alcoholic liver disease [4]. Glomerular IgA deposition is also common in celiac disease, occurring in as many as 1/3 of patients with celiac disease [3]. There have been a few case reports of IgAN associated with hepatitis A infection, but no case has been reported of IgAN associated with HEV [6]. Unlike hepatitis B & C, renal involvement in patients with HEV infection is extremely rare and has only been reported in immunocompromised individuals. HEV-induced membranoproliferative glomerulonephritis has been reported in an immunocompetent individual [7]. Hepatitis E genotype 3 has been reported to cause several glomerular manifestations, including relapse of IgA nephropathy, but only in recipients of solid organ transplantation who were immunocompromised [7]. Kamar et al. [8] 2012 reported a “relapse of IgAN” in solid organ recipients during HEV infection. There was no de-novo case of IgAN in this cohort or any other immunocompetent patient ever reported in the past that has been associated with or exacerbated by acute HEV infection. 

While he was being treated for hepatitis E infection, one-plus proteinuria on initial dipstick urine analysis was thought to be due to increased urinary concentration as indicated by high urine specific gravity. Urinary protein creatinine ratio was not done at that time. Another likely explanation of 1+ proteinuria on dipstick could be due to the presence of a milder form of IgA nephropathy prior to HEV infection. Moreover, the presence of some segmental and global glomerular sclerosis may also raise the suspicion that IgAN may have been present for some time, and association with acute hepatitis may have been just by chance. So, the possibility of ongoing IgAN exacerbated by acute HEV infection cannot be ruled out either. But the presence of only mild interstitial fibrosis and tubular atrophy favors the recent onset of IgAN and its association with HEV infection. 

Post-infectious glomerulonephritis and IgAN have been reported in the literature. In fact, IgA-dominant acute post-infectious glomerulonephritis is an important differential diagnosis in which IgA is the dominant immunoglobulin in glomerular deposits [9]. It usually occurs in association with staphylococcal infection but not with hepatitis. Though it is difficult to prove mild interstitial fibrosis and tubular atrophy or global sclerosis in two glomeruli with no prior history of glomerular disease, the onset of nephrotic syndrome immediately after HEV infection and complete remission after resolution of infection, albeit with steroids, all support the notion that IgAN in our patient is most likely associated with HEV infection if not directly caused by it. 

The exact mechanism of this association is not clear, but in patients with hepatitis and liver disease, impaired removal of IgA-containing complexes by the Kupffer cells in the liver is the likely reason for IgA deposition in the kidney. The mechanism of glomerular injury due to HEV is not well understood but is considered to be immune complex-mediated like HBV & HCV [10, 11]. 

In addition to supportive therapy for nephrotic syndrome, immunosuppression with steroids was initiated since the acute hepatitis E infection had recovered and patient was thought to be at high-risk for IgAN progression because of elevated creatinine at presentation and nephrotic syndrome with hypoalbuminemia. The patient responded well to the regimen, and complete remission was achieved after 6 months of treatment. Up to the reporting of this case, the patient was still on low-dose statins, ARBs, and prednisone (10 mg/day) and continued to show improvement on tapering dosages of steroids without any relapses at 6 months. This excellent response could be due to immunosuppression and/or recovery after acute hepatitis E infection. This case report highlights the need of further scientific research to identify the exact mechanism of IgAN in acute HEV infection. 

In conclusion, we presented a first biopsy-proven case report of a patient with IgAN associated with acute HEV infection. IgAN in this patient manifested with nephrotic syndrome, gross hematuria, and acute kidney injury. 

## Funding 

The authors received no financial support for the research, authorship, and/or publication of this article. 

## Conflict of interest 

The authors have no conflict of interest to disclose.
